# Diagnostic Models for Predicting Follicular Thyroid Carcinomas Using Circulating Plasma MicroRNAs

**DOI:** 10.3390/cancers17213401

**Published:** 2025-10-22

**Authors:** Sin Woo Kang, Ji Min Kim, Sung-Chan Shin, Yong-Il Cheon, Bo Hyun Kim, Mijin Kim, Sang Soo Kim, Byung-Joo Lee

**Affiliations:** 1 Department of Otorhinolaryngology-Head and Neck Surgery, College of Medicine, Pusan National University and Biomedical Research Institute, Pusan National University Hospital, Busan 49241, Republic of Korea; 2 Pusan National University Medical Research Institute, Pusan National University School of Medicine, Pusan National University, Yangsan 50612, Republic of Korea; 3 Department of Internal Medicine, College of Medicine, Pusan National University and Biomedical Research Institute, Pusan National University Hospital, Busan 49241, Republic of Korea; 4 Department of Otorhinolaryngology-Head and Neck Surgery, Good Gang-An Hospital, Busan 48265, Republic of Korea

**Keywords:** circulating miRNA, diagnosis, follicular thyroid carcinoma

## Abstract

Background: Follicular thyroid carcinoma (FC) accounts for 10–15% of all thyroid cancers. FC is challenging to diagnose using fine-needle aspiration (FNA), making diagnostic thyroidectomy the standard approach. Recent studies have explored the use of circulating microRNAs for thyroid cancer diagnosis. This study evaluated the diagnostic value of circulating miRNAs in plasma for FC to potentially reduce unnecessary thyroidectomies and repeat invasive procedures. Methods: This study was a retrospective observational study, and included consecutively selected 90 patients who underwent thyroidectomy at Pusan National University Hospital between January 2013 and February 2024 and were diagnosed with FC (49 patients) or follicular thyroid adenoma (FA) (41 patients) on final histopathology. Of these, 58 patients were enrolled in the utility assessment and 32 patients were included in the validation test. Among the 58 patients included in the utility assessment, microarray analysis was conducted on 15 patients who were randomly selected to identify novel plasma miRNAs. Next, TaqMan qRT-PCR was performed to evaluate the diagnostic utility of five plasma miRNAs and to develop a predictive model capable of predicting FC from FA using logistic regression as the utility assessment on 58 patients. Finally, in the validation test, TaqMan qRT-PCR and statistical analysis were conducted again on 32 patients and the constructed predictive models, verifying the accuracy of the predictive model. Results: Using microarray analysis, a novel miRNA, miR-6085, was identified for its distinguishing capability between FC and FA. In the utility assessment, miR-6085, miR-146b-5p, miR-221, and miR-222 were significantly upregulated in the FC group. A predictive model combining these four miRNAs showed strong diagnostic value for FC, with an AUC of 0.928 (0.843, 1.000), sensitivity of 94.7% (84.2, 100), specificity of 86.4% (68.2, 100). The accuracy of this model was 76.2% (52.8, 91.8) in the validation test. Conclusions: A model combining four miRNAs (miR-6085, miR-146b-5p, miR-221, and miR-222) demonstrated high sensitivity, specificity, and accuracy, suggesting that it could be a useful tool for differentiating FC from FA.

## Simple Summary

Preoperative differentiation between follicular adenoma and carcinoma remains challenging by routine cytology. We identified miR-6085 as a novel circulating plasma biomarker for follicular thyroid carcinoma. Building on this, we developed a four-marker model (miR-6085, miR-146b-5p, miR-221, miR-222) that showed strong diagnostic performance in the utility assessment (AUC 0.928; sensitivity 94.7%; specificity 86.4%), and its ability to distinguish carcinoma from adenoma was confirmed in a validation set with an accuracy of 76.2%. These results support the potential of circulating miRNA-based models to reduce unnecessary thyroidectomies and other invasive procedures.

## 1. Introduction

Thyroid cancer is one of the most common cancers worldwide, with an increasing incidence worldwide [1,2]. Follicular thyroid carcinoma (FC) accounts for 10–15% of thyroid cancers and exhibits higher metastasis, mortality, and recurrence rates than papillary thyroid carcinoma (PTC) [3,4]. PTC, the most common type, is accurately diagnosed preoperatively using ultrasound and fine-needle aspiration (FNA). Unlike PTC, FC has no typical ultrasound findings, making it difficult to differentiate FC from FA by preoperative ultrasound examination. Also, there are no available specific preoperative molecular markers that can differentiate FC from FA. In addition, when US-FNA is performed for diagnosis, it is often diagnosed as Bethesda category IV (follicular neoplasm or suspicious for follicular neoplasm). When FNA results are classified as Bethesda category IV, diagnostic thyroidectomy is often recommended. However, the incidence of malignant tumors after surgery is only 23–34%, so unnecessary surgery is performed in more than two-thirds of patients [5]. If malignant follicular tumors can be predicted preoperatively, diagnostic thyroidectomy could be avoided in benign follicular neoplasms, while early intervention for high-risk cases could improve patient outcomes and reduce healthcare costs.

MicroRNAs (miRNAs) are small non-coding RNAs regulating gene expression. They influence immune responses, hormone secretion, circadian rhythms, cell proliferation, and apoptosis [6,7]. MiRNAs are abnormally expressed in various cancers, including leukemia, prostate, breast, bladder, and colorectal cancers [8]. Research explores the use of circulating plasma miRNAs for cancer diagnosis, offering a non-invasive alternative to FNA or core needle biopsy (CNB) [9,10,11].

In patients with PTC, various miRNAs are either upregulated or downregulated in tumor tissues and body fluids, and numerous studies have explored their use in diagnosing PTC and predicting its prognosis [12,13]. In case of FC, studies have also analyzed miRNA expression in post-surgical tumor tissue, with increased expression of miRNAs such as miR-146b, miR-197, miR-221, and miR-222 [14,15,16,17]. However, comprehensive studies of plasma miRNAs in FC patients are lacking. This study aimed to investigate the potential of plasma miRNA profiling to distinguish follicular adenoma (FA) from follicular carcinoma (FC), and to identify novel miRNAs through microarray analysis that may serve as potential biomarkers for predicting FC. Furthermore, we sought to validate the accuracy of the developed prediction model through a validation test.

## 2. Materials and Methods

### 2.1. Patients

**Figure 1:**
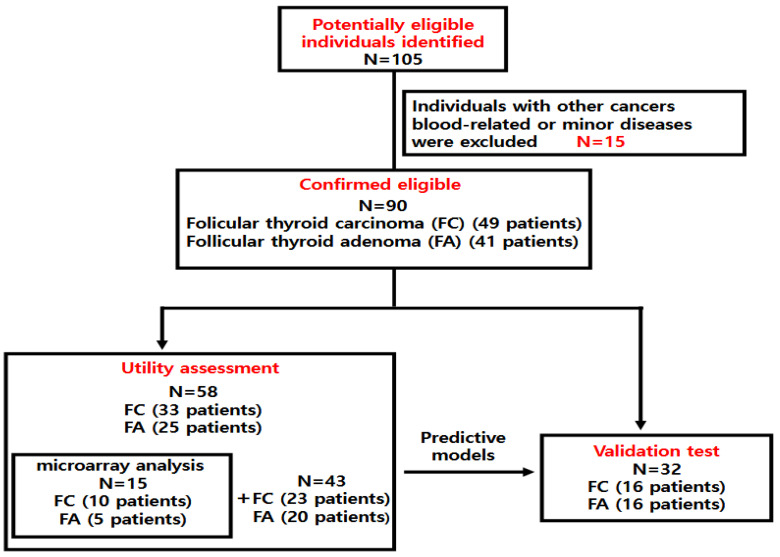
Participant flow diagram. Of the 105 patients diagnosed with FA or FC, 15 were excluded. Utility assessment of plasma microRNAs was performed on 58 subjects, including 15 subjects who underwent microarray analysis. A validation test was conducted to evaluate the accuracy of the predictive model for 32 patients.

This study was a retrospective observational analysis. One hundred five patients who underwent thyroidectomy at the Department of Otorhinolaryngology, Pusan National University Hospital, between January 2013 and February 2024 were consecutively enrolled in this study. All patients were diagnosed with FC or FA based on final histopathological examination. Fifteen individuals with other cancers, blood-related or minor diseases were excluded. We analyzed plasma circulating microRNA from 90 patients diagnosed with FC or FA to identify novel microRNA biomarkers predictive of FC and to develop diagnostic prediction models. The study was conducted in three sequential phases: microarray, utility assessment, and validation test. To minimize selection bias, patients were enrolled consecutively and then randomly assigned to each cohort group. Blood samples were collected from patients who provided written informed consent before surgery, and no hemolyzed samples were included in the analysis. This cross-sectional study was approved by the Institutional Review Board of the Pusan National University Hospital (2307-014-129). Plasma samples were collected immediately prior to surgery and stored at −80 °C in an ultra-low temperature freezer, managed by Biobank of Pusan National University Hospital. Among the 90 patients, there were 49 patients with FC and 41 patients with FA. Fifty-eight were enrolled in a study to evaluate the utility of plasma miRNAs for distinguishing between FA and FC as the utility assessment. Among the 58 patients included in the utility assessment, microarray analysis was conducted on 15 individuals who were randomly selected to identify novel plasma miRNAs prior to utility assessment. In addition, other 32 patients were enrolled in a study to validate the accuracy of the constructed predictive models that were determined to be useful (Figure 1). There were no statistical differences in sex ratio, age and tumor size among the three cohorts (Microarray, Utility assessment, validation test). Detailed epidemiological information for each study group is presented in Table 1 and Table 2.

### 2.2. Selection of MicroRNA

Microarray analysis was performed on 15 patients–five with FA, five with minimally invasive follicular carcinoma (MI-FC), and five with widely invasive follicular carcinoma (WI-FC) to identify novel microRNAs that differentiate between benign and malignant follicular tumors. We found that miR-6085 was significantly overexpressed in serum of patients with follicular carcinoma compared to that of follicular adenoma via microarray. Since there are few studies on circulating plasma miRNAs that are upregulated in FC, we selected miRNAs already known to be upregulated in follicular carcinoma tissues in previous studies, including miR-126-3p, miR-146b-5p, miR-221, and miR-222 [14,15,16,17]. Even though miR-126-3p, miR-146b-5p, miR-221, miR-222 showed no significant difference in microarray, we included these miRNAs to improve the capability of predictive models. Using this new miRNA (miR-6985), along with miR-126-3p, miR-146b-5p, miR-221, and miR-222, we analyzed the five plasma miRNAs from 58 patients (25 with FA and 33 with FC) using the TaqMan method. Following laboratory experiments, including microarray and qRT-PCR, were conducted in a blind manner to reduce information bias

### 2.3. Microarray

The RNA purity and integrity were assessed using an ND-2000 Spectrophotometer (NanoDrop, Wilmington, DE, USA) and an Agilent 2100 Bioanalyzer (Agilent Technologies, Palo Alto, CA, USA). The Affymetrix Genechip miRNA 4.0 array procedure (Affymetrix, Santa Clara, CA, USA) was performed following the manufacturer’s instructions. RNA samples (1000 ng) were labeled using the FlashTag Biotin RNA Labeling Kit (Genisphere, Hatfield, PA, USA). The labeled RNA was quantified, fractionated, and hybridized to the miRNA microarray according to standard procedures provided by the manufacturer. The labeled RNA was heated at 99 °C for 5 min, followed by 45 °C for 5 min. Subsequently, RNA-array hybridization was performed with agitation at 60 rotations per minute for 16 h at 48 °C using an Affymetrix 450 Fluidics Station (Affymetrix, Inc., Santa Clara, CA, USA). The chips were washed and stained using a GeneChip Fluidics Station 450 (Affymetrix). Chips were scanned using an Affymetrix GCS 3000 scanner (Affymetrix, Inc., Santa Clara, CA, USA). Signal values were determined using the Affymetrix GeneChip Command Console software 4.0.

### 2.4. Microarray Raw Data Preparation and Statistical Analysis

Raw data were automatically extracted using the Affymetrix data extraction protocol implemented in the Affymetrix GeneChip Command Console Software (AGCC). Subsequent processing, including the import of CEL files, miRNA expression analysis using the RMA + DABG-All method, and export of results, was performed using the Affymetrix^®^ Power Tools (APT) Software 4.0. The array data were filtered to include only probes annotated for the species of interest. Comparative analysis between the test and control samples was conducted using an independent *t*-test and fold-change analysis, with the null hypothesis positing no difference between the groups. The false discovery rate (FDR) was controlled by adjusting the *p*-values using the Benjamini–Hochberg algorithm. All statistical analyses and visualizations of differentially expressed genes were conducted using R software (version 3.3.2; R Foundation for Statistical Computing, Vienna, Austria).

### 2.5. RNA Isolation

Total RNA was isolated from 800 µL of plasma using the mirVana PARIS™ kit (Thermo Fisher Scientific, Waltham, MA, USA), following the manufacturer’s guidelines. In brief, 400 µL of plasma was combined with an equal volume of 2× denaturing solution at 16 °C and incubated on ice for 5 min to facilitate miRNA preservation. Subsequently, 650 µL of acid-phenol was added, and the sample was vortexed vigorously for 10 s. After centrifugation at 10,000× *g* for 10 min at 4 °C, the upper aqueous layer was carefully transferred to a fresh tube. Ethanol was then added at a ratio of 1.25:1 (ethanol/supernatant), mixed, and the sample was incubated for 5 min at 16 °C. A 600 µL aliquot of the mixture was loaded onto a filter cartridge inserted in a collection tube and centrifuged at 10,000× *g* for 30 s at 4 °C. The column was sequentially washed: first with 600 µL of wash solution 1 (centrifuged at 10,000× *g* for 30 s at 4 °C), and then twice with 500 µL of wash solution 2/3 (the first spin for 30 s, and the second for 1 min under the same conditions). Finally, the cartridge was relocated to a new tube, and total miRNA was eluted with 50 µL of elution solution. The eluate was collected by centrifugation at 10,000× *g* for 1 min at 4 °C and stored at −80 °C until further use

### 2.6. Reverse Transcription Reaction

Complementary DNA (cDNA) was synthesized from isolated RNA using the TaqMan MicroRNA Reverse Transcription Kit (Applied Biosystems, Foster City, CA, USA), in accordance with the manufacturer’s protocol. Each RT reaction was prepared in a total volume of 15 µL, comprising 5 µL of RNA template, 1.5 µL of 10× RT buffer, 0.2 µL of RNase inhibitor (20 U/µL), 1 µL of reverse transcriptase (50 U/µL), and 0.15 µL of 100 mM dNTP mix. To enable specific reverse transcription of target miRNAs (miR-6085, miR-146b-5p, miR-221, miR-222, miR-3196 and miR-6126 and miR-191), 3 µL of 5× miRNA-specific stem-loop primers were included, along with 4.15 µL of nuclease-free water to complete the final volume. Thermal cycling conditions for RT were as follows: 30 min at 16 °C, followed by 30 min at 42 °C, and enzyme inactivation at 85 °C for 5 min.

### 2.7. Quantitative Real-Time PCR

Quantification of miRNA expression was performed via real-time PCR using the 7900HT Fast Real-Time PCR System (Applied Biosystems, Foster City, CA, USA) in combination with the TaqMan^®^ Universal PCR Master Mix (Thermo Fisher Scientific). Each reaction was prepared in a total volume of 20 µL, consisting of 2 µL of synthesized cDNA, 10 µL of PCR Master Mix, 1 µL of 20× TaqMan miRNA-specific assay (for miR-6085, miR-126-3p, miR-146b-5p, miR-221, miR-222, and miR-191), and 7 µL of nuclease-free water. The PCR protocol included an initial enzyme activation step at 95 °C for 10 min, followed by 40 amplification cycles of denaturation at 95 °C for 15 s and annealing/extension at 60 °C for 1 min. Relative expression levels of target miRNAs were analyzed using the comparative threshold cycle (ΔCt) method. Among the tested miRNAs, miR-191, which exhibited consistent expression across all samples, was employed as the internal control for normalization.

### 2.8. Statistics Analysis

Statistical analyses were performed using GraphPad Prism software version 10 (GraphPad Prism, Boston, MA, USA). Continuous variables were summarized as mean ± standard deviation or median (interquartile range), depending on normality. No continuous variables were categorized for analysis; all were used in their original continuous form. Unless otherwise stated, all quantitative data are reported as the mean standard error of the mean or standard deviation for all samples. Mann–Whitney U test was used to determine significant differences between groups, with *p* < 0.05 considered statistically significant (SD). To compare the expression levels of each gene, ROC curves of 58 TaqMan RT-PCR patients were constructed, and the AUC was calculated. Subsequently, considering the expression levels of all the miRNAs, we created the most predictive model to distinguish FC. We also constructed a combination predictive model using multiple significant microRNAs to improve the capability of single microRNA model. Finally, we evaluated the accuracy of the constructed predictive models using four significant microRNAs (miR-6085, miR-146b-5p, miR-221, miR-222) by conducting a verification examination of 32 patients (16 with FA and 16 with FC) who were not included in model construction (Table 1). Risk over time was not evaluated as this study focused on diagnostic model development and not on prognostic outcomes. Statistical analyses and model development were conducted with the assistance of the Department of Biostatistics at the Pusan National University Hospital Research Institute of Convergence Biomedical Science.

## 3. Result

### 3.1. Microarray Between FC and FA

**Figure 2:**
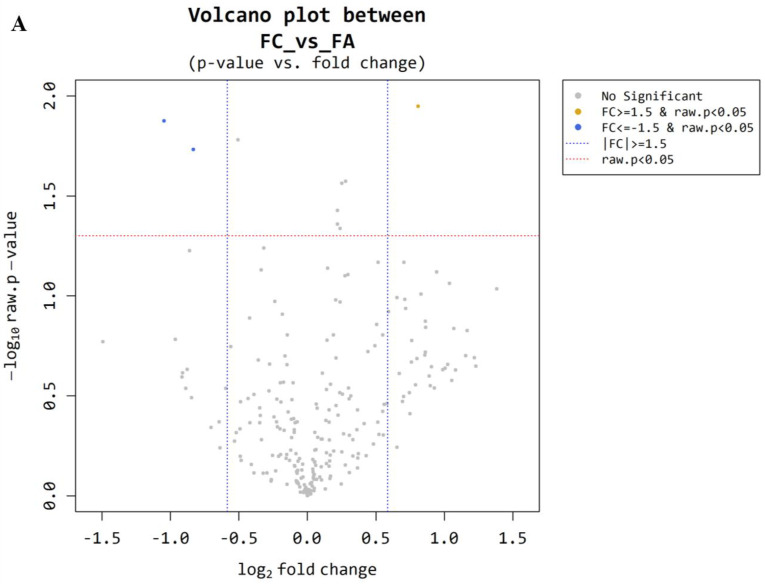

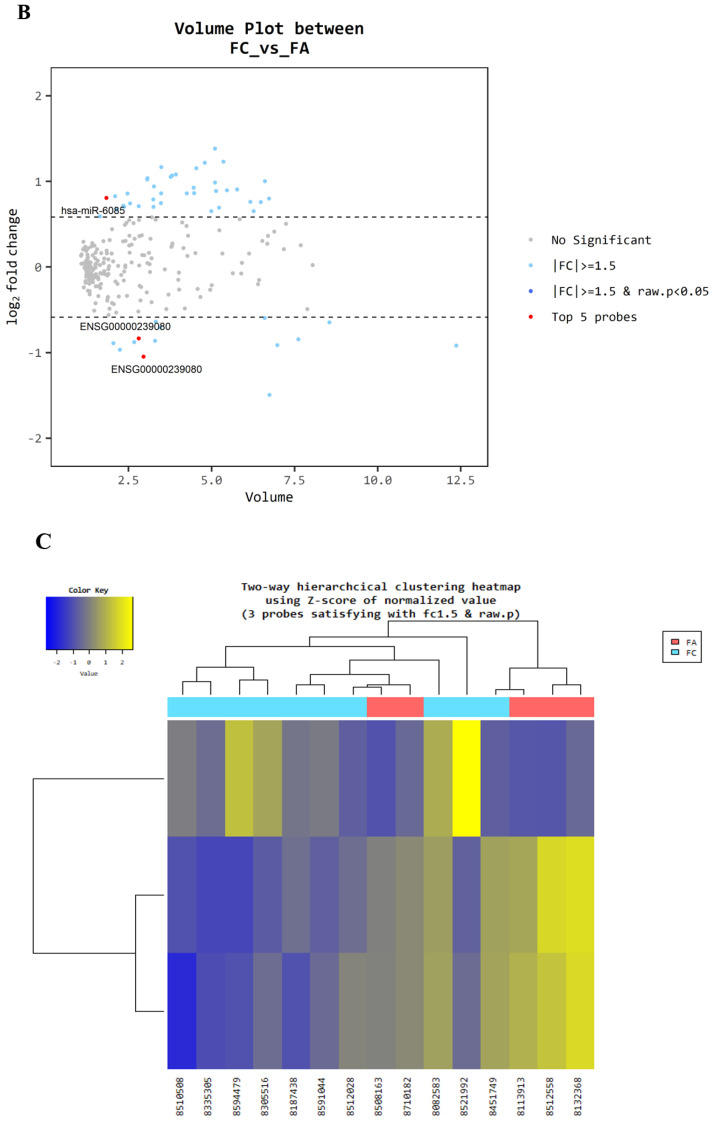
Microarray results for follicular thyroid adenoma and carcinoma. (**A**) Volcano plot. The *x*-axis represents the log_2_ fold change, and the *y*-axis shows the −log_10_ (raw *p*-value), indicating statistical significance. Each dot represents a probe (e.g., gene or transcript), and is color-coded as follows: Orange dots: Significantly upregulated in follicular thyroid carcinoma (FC) compared with follicular thyroid adenoma (FA) (fold change ≥ 1.5 and raw *p* < 0.05). Sky blue dots: Significantly downregulated in FC (fold change ≤ −1.5 and raw *p* < 0.05). Vertical dotted blue lines indicate the fold change threshold at log_2_ (±1.5), and the horizontal red dotted line marks the *p*-value significance cutoff at raw *p* = 0.05. (**B**) Volume plot. The *x*-axis represents the Volume (likely referring to expression abundance or a related metric), and the *y*-axis represents the log_2_ fold change between the two groups. Red dots: Dashed horizontal lines indicate log_2_ fold change thresholds of ±1 (corresponding to fold change of ~2). Labeled points highlight selected genes (hsa-miR-6085, ENSG00000239080). (**C**) Hierarchical clustering heat map. The degree of similarity among the plasma miRNAs was visualized in 2D space using the two components that best explained the variance in the entire dataset: fold change (*x*-axis) and raw *p*-value (*y*-axis). This visualization allowed us to identify outlier samples and assess whether sample groups exhibited similar expression patterns.

Microarray analysis was conducted on plasma samples from 5 patients in the benign control group (FA) and 10 patients in the malignancy group (FC). Using a volcano and volume plot (Figure 2A,B), three miRNAs with more than 1.5-fold differential expression between the two groups were identified (*p* < 0.05). Hierarchical clustering was performed, and a heatmap was created to visualize the results (Figure 2C). Three miRNAs were identified, including one novel miRNA (miR-6085, miRBase Accession Number: MI0020362, AAGGGGCUGGGGGAGCACA), and two unannotated miRNAs (ENSG00000239080; probe ID; 20533711, ENSG00000239080; probe ID; 20533712). A novel miRNA, miR-6085, was upregulated in the FC group compared to the positive group (*p* < 0.05) (Table 3).

### 3.2. Plasma miRNAs TaqMan (qRT-PCR) Assay Between FC and FA (Utility Assessment)

A TaqMan assay (qRT-PCR) was performed on 58 patients (33 FC patients and 25 benign patients) to investigate miRNAs identified in the previous plasma miRNA microarray (miR-6085) and four miRNAs previously reported to show differential expression in FC tissues (miR-126-3p, miR-146b-5p, miR-221, and miR-222) as a utility assessment. Differences in miRNA expression between the malignant experimental group and benign control group were validated using a Mann–Whitney U test and *t*-test on fold changes.

**Figure 3:**
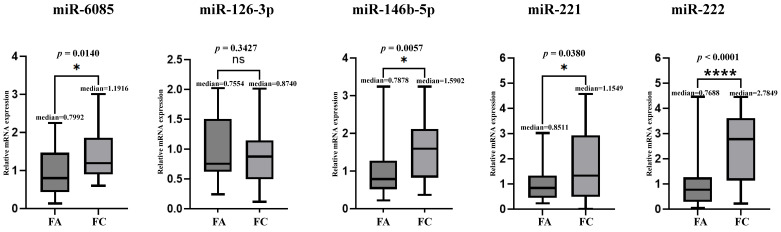
Relative miRNA expression of follicular thyroid adenoma and carcinoma. This graph compares the fold changes in the five miRNAs between follicular thyroid carcinoma (FC) and follicular thyroid adenoma (FA). * denote statistical significance: * indicates *p* < 0.05, and **** indicates *p* < 0.0001. Significant upregulation of miR-6085, miR-146b, miR-221, and miR-222 expression was also observed.

The results demonstrated significant differential expression of miR-6085, miR-146b-5p, miR-221, and miR-222 between the FC and Benign groups, whereas miR-126-3p did not exhibit significant difference. Even after excluding 15 patients who underwent microarray analysis from the 58 patients in the utility assessment, the results remained the same. Compared to the benign control group, the relative fold change in the FC patient group was as follows: miR-6085 was up-regulated by 1.49 ± 2.14, miR-126-3p by 0.86 ± 0.23, miR-146b-5p by 1.92 ± 2.23, miR-221 by 1.60 ± 3.10, and miR-222 by 3.62 ± 5.15 (95% CI) (Figure 3).

### 3.3. Prediction of Malignancy of FC by ROC Curve of Plasma MicroRNA

**Figure 4:**
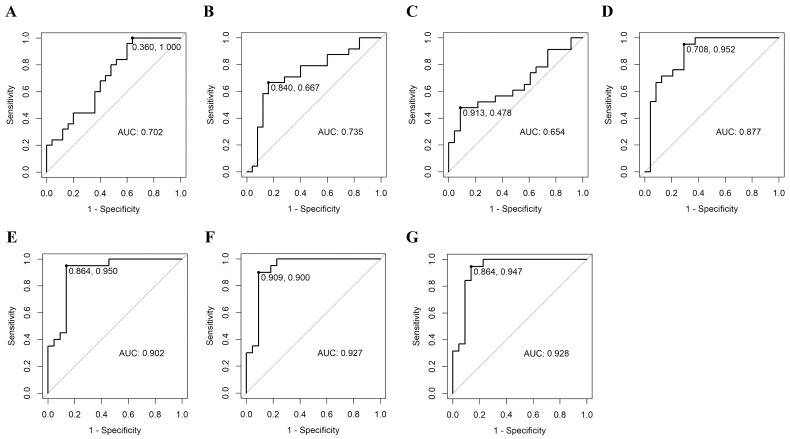
Diagnostic models for predicting follicular thyroid carcinoma. (**A**): model 1-1 (miR-6085), (**B**): model 1-2 (miR-146b-5p), (**C**): model 1-3 (miR-221), (**D**): model 1-4 (miR-222), (**E**): model 2-1 (miR-221 + miR-222), (**F**): model 3-1 (miR-6085 + miR-221 + miR-222), (**G**): model 4 (miR-6085 + miR-146b-5p + miR-221 + miR-222). Model 1-4 (miR-222) was the most predictive model among 4 single miRNA models. The model combining all miRNAs (Model 4) showed the most powerful ROC curve for predicting follicular thyroid carcinoma. The predictive power tended to increase when more miRNAs were included in the model.

We developed a predictive model for FC malignancy using four plasma miRNAs (miR-6085, miR-146b-5p, miR-221, and miR-222) that significantly differentiated FC from benign cases in the TaqMan assay. A model was constructed using ROC curve analysis to create the most efficient predictive model by combining multiple miRNAs. Among the predictive models using single miRNA, the model of miR-222 demonstrated highest AUC of 0.877 ± 0.106 (95% CI) with good sensitivity of 0.952. For the models combining two miRNAs, miR-221 + miR-222 model achieved the highest AUC of 0.902 ± 0.098. Among the models combining three miRNAs, the miR-6085 + miR-221 + miR-222 combination produced a higher AUC of 0.927 ± 0.087. The model combining the four miRNAs (miR-6085 + miR-146b-5p + miR-221 + miR-222) achieved the highest AUC (0.928 ± 0.085) with excellent sensitivity (0.947), specificity (0.864), and a BIC of 44.77, indicating strong predictive performance and model fit. There was a trend toward an increasing AUC as more miRNAs were included in the analysis. The ROC curves, AUC, sensitivity, specificity, cut-off values, and the model formulas are detailed in Table 4 and Figure 4.

### 3.4. Microarray Between MI-FC and WI-FC

**Figure 5:**
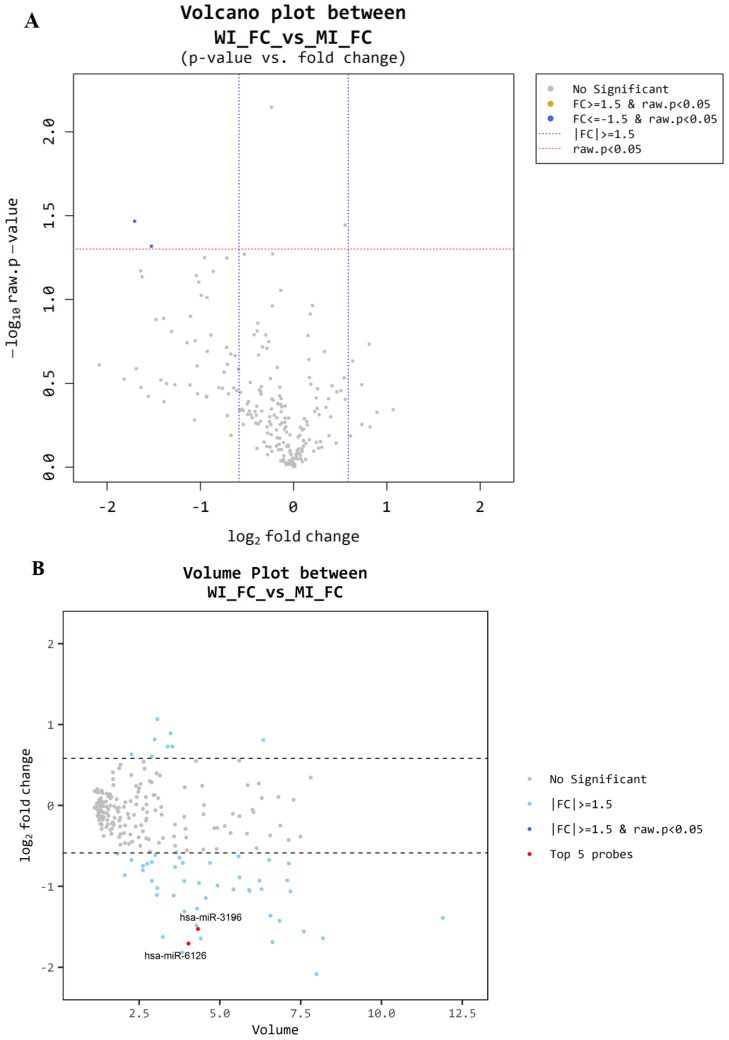

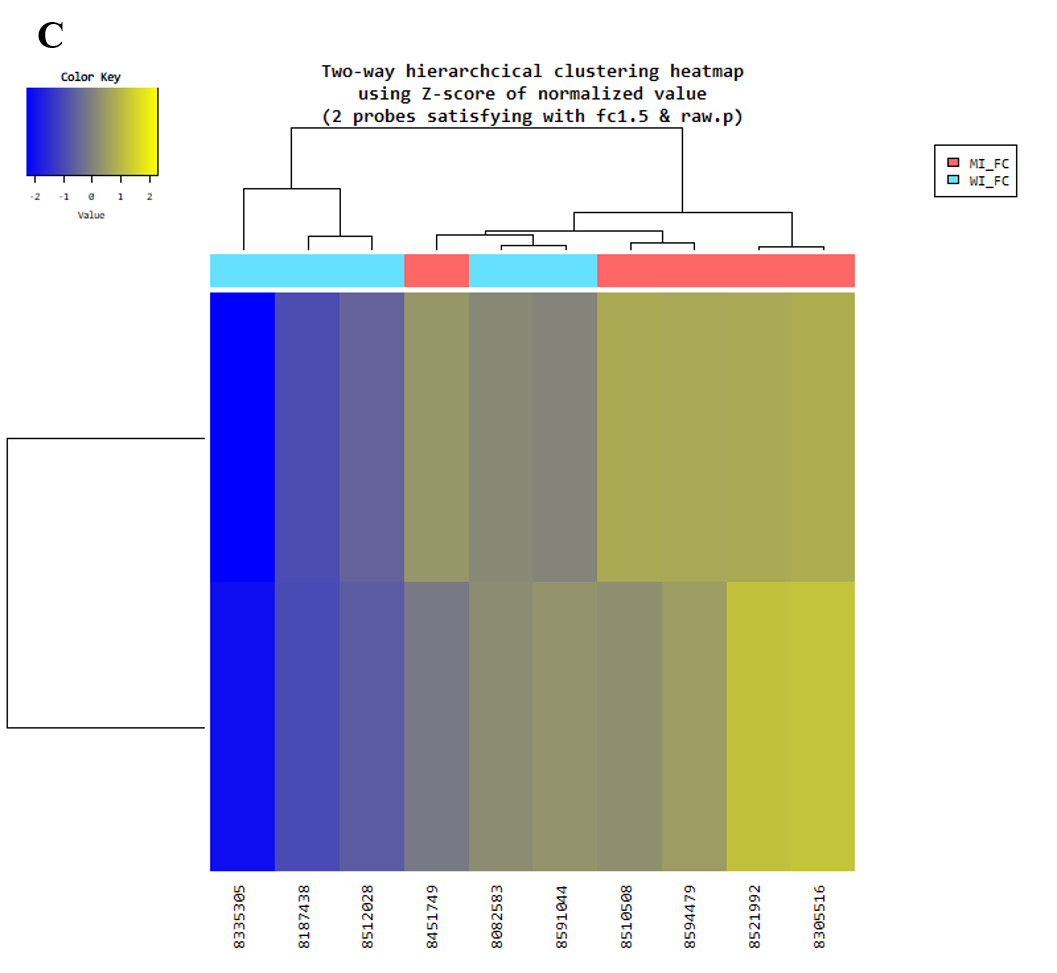
Microarray results for minimally invasive follicular carcinoma and widely invasive follicular carcinoma. (**A**) Volcano plot. Blue dots: Significantly downregulated in widely invasive follicular carcinoma (WI FC) compared with minimally invasive follicular carcinoma (MI FC) (fold change ≤ −1.5 and raw *p* < 0.05). (**B**) Volume plot. Red dots: Dashed horizontal lines indicate log_2_ fold change thresholds of ±1 (corresponding to fold change of ~2). Labeled points highlight selected genes (hsa-miR-3196, hsa-miR-6126). (**C**) Hierarchical clustering heat map.

Among the 10 patients with FC, microarray analysis was also performed on plasma samples from each of the five patients with WI-FC and MI-FC. Differential expression analysis using volcano and volume plots (Figure 5A,B) revealed two miRNAs exhibiting greater than 1.5-fold changes in expression between the two groups, with statistical significance (*p* < 0.05). We performed hierarchical clustering and created a heatmap to visualize the results (Figure 5C). Two miRNAs, miR-3196 (miRBase Accession Number: MI0014241, CGGGGCGGCAGGGGCCUC) and miR-6126 (miRBase Accession Number: MI0021260, GUGAAGGCCCGGCGGAGA), were identified as significantly downregulated in the WI-FC group relative to the MI-FC group.

### 3.5. Microarray and TaqMan (qRT-PCR) Assay Between MI-FC and WI-FC

**Figure 6:**
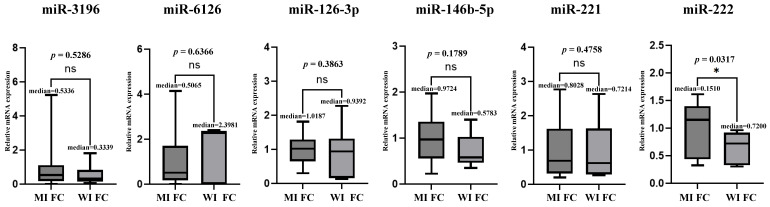
Relative miRNA expression of minimally invasive follicular carcinoma and widely invasive follicular carcinoma. This graph compares the fold changes in the five miRNAs between widely invasive follicular carcinoma (WI-FC; 8 patients) and minimally invasive follicular (MI-FC; 25 patients). * denote statistical significance: * indicates *p* < 0.05. No significant differences in the expression levels were observed for miR-3196, miR-6126, miR-146b-5p and miR-221.

For each patient with WI-FC (n = 8) and MI-FC (n = 25), the TaqMan qRT-PCR assay was performed to examine the expression levels of miR-3196 and miR-6126, as well as miRNAs previously reported to be upregulated in FC (miR-126-3p, miR-146b-5p, miR-221, and miR-222). The results showed that miR-222 was downregulated in the WI-FC group compared to the MI-FC group. However, no statistically significant differences were observed in the expression levels of the remaining five miRNAs between the two groups (Figure 6). In the microarray analysis, miR-3196 and miR-6126 were downregulated compared to those in MI-FC. Decreasing plasma miRNA levels have limitations in their use as biomarkers for clinically identifying cancer. Moreover, due to the small number of patients with WI-FC and the lack of significance of miRNAs in the TaqMan assay, further predictive examinations were not conducted.

### 3.6. Validation Test of Predictive Models

A validation examination was conducted in 32 patients (16 with FC and 16 with benign) using a previously developed predictive model of FC. None of the 32 patients were included in the initial cohort of 58 patients, from whom miRNA expression levels were measured using TaqMan qRT-PCR. miRNA expression levels in these 32 patients were measured in the same manner and applied to the predictive model to assess the accuracy, sensitivity, and specificity of FC diagnosis. Since the percentage of malignant patients who were enrolled in validation test was 50% (16 patients with FC, 16 patients with benign), a cutoff threshold of 50% was used. Cases were classified as malignant if the predicted likelihood of malignancy calculated by the statistical model exceeded 50%. Accuracy was determined by calculating the percentage of cases in which the model correctly identified malignant and benign conditions.

The results showed that models using single miRNAs or two-miRNA combinations had low accuracy below 0.7, whereas models combining three or four miRNAs demonstrated higher accuracy. Among the predictive models of single microRNA, miR-222 was the most accurate model, showing accuracy of 0.679, sensitivity of 0.769, specificity of 0.600. The model combining miR-6085, miR-221 and miR-222 yielded an accuracy of 0.739, sensitivity of 0.727, specificity of 0.750. The combined model of all four miRNAs (miR-6085, miR-146b-5p, miR-221, miR-222) was selected as the most accurate model for validation, accuracy of 0.762, sensitivity of 0.700, specificity of 0.818. The accuracy, sensitivity, and specificity of each model are listed in Table 5.

## 4. Discussion

When FC is suspected via ultrasound, FNA, or CNB, diagnostic thyroidectomy is the standard diagnostic method. The 2015 American Thyroid Association guidelines recommend surgery for follicular neoplasms >1 cm with extrathyroidal extension or lymph node involvement. Molecular testing may supplement clinical and ultrasound findings [18]. For Bethesda category IV cases, malignancy rates post-surgery range from 23–34% [5]. Since 60% of adults and 85% of children with follicular neoplasms undergo thyroidectomy, predicting malignancy preoperatively could reduce unnecessary surgeries, patient burden, and healthcare costs [19]. This study aimed to develop a predictive model for FC using plasma miRNAs as a supplementary diagnostic tool.

MiRNA research in thyroid cancer has mainly focused on PTC. Meta-analyses report overexpression of miR-146b-5p, miR-221, miR-222, and miR-181b in PTC tissues, with miR-146b-5p, miR-221, and miR-222 upregulated in other thyroid cancers [20]. Circulating miRNAs such as miR-146b-5p (AUC of 0.900 (95% CI, 0.833–0.966)), miR-221 (AUC of 0.650 (95% CI, 0.524–0.776), miR-222 (AUC of 0.821 (95% CI, 0.724–0.917)) have been studied in PTC, but FC research remains limited [12]. FC studies have identified miR-146b-5p, miR-197, miR-221, miR-222, and miR-346 as potential tumor tissue biomarkers, with miR-197 and miR-346 specifically upregulated in FC [14,15,16,17,20]. MiR-21 (upregulated in FC) and miR-181a (upregulated in PTC) have shown differential expression between FC and PTC in peripheral blood samples, with sensitivity of 100% (95% CI, 81.5–100), specificity of 77% (95% CI, 58.9–90.4), though their combined analysis [21]. This study aimed to compare miRNA expression in benign and malignant follicular thyroid tumors. In this study, we mainly analyzed five miRNAs (miR-6085, miR-126-3p, miR-146b-5p, miR-221, miR-222) and used four significant miRNAs in constructing the predictive model of FC.

MiR-146b-5p, miR-221, and miR-222 are well-known miRNAs that are commonly upregulated in all types of thyroid cancer tissues and are overexpressed in the peripheral blood samples of patients with PTC [20]. Zhang et al. proposed a model combining the expression levels of miR-222, miR-221, miR-146b, and miR-21 in the serum with ultrasound results to diagnose PTC with high accuracy (AUC = 0.971) [12]. miR-146b-5p, located on chromosome 10q24.32, targets 34 genes involved in cell proliferation, differentiation, apoptosis, cell cycle, and signaling [22]. miR-221 and miR-222, which are known for their consistent upregulation in thyroid cancers, have similar sequences and are clustered on the X chromosome. These miRNAs target c-KIT and p27, which influence the cell cycle and contribute to thyroid cancer development [23]. miR-6085 was a novel miRNA that distinguished FC and FA on microarray. Therefore, it was used in the diagnostic model to predict FC. miR-6085, a microRNA rarely mentioned in previous thyroid cancer studies, was found to inhibit AIFM2 and GPX4 protein expression, mediate cell ferroptosis in hepatocellular carcinoma (HCC) [24], and target the GDF11 protein, which is involved in cell apoptosis and oxidative stress in human brain microvascular endothelial cells of stroke patients [25]. In addition, Liu et al. reported that miR-6085 participated in the circDLGAP4/miR-6085/GDF11 pathway and contributed to endothelial damage under ischemic conditions. Since vascular invasion is a key histopathological feature used to diagnose follicular thyroid carcinoma, this finding raises the possibility that miR-6085 could also be mechanistically linked to vascular invasion in thyroid follicular neoplasms [25]. This study did not analyze the mechanisms by which miR-6085 contributes to FC development, which is a limitation that warrants further investigation in future research.

Circulating miRNAs often mirror tumor tissue expression, but not always [26,27]. This study’s strength lies in analyzing plasma miRNAs from patients diagnosed with follicular neoplasm via FNA or CNB, rather than post-surgical tissues, to identify FC biomarkers. In addition to miR-146b-5p, miR-221, and miR-222, microarray analysis identified miR-6085 as a potential biomarker. This study also assessed the predictive value of miRNAs for FC malignancy using ROC curves. A predictive model combining miR-6085, miR-146b-5p, miR-221, and miR-222 showed high diagnostic value, with an AUC of 0.928, sensitivity of 0.947, specificity of 0.864 in utility test, and accuracy of 0.762 in validation testing.

A recent meta-analysis on the diagnosis of PTC using circulating miRNAs found that across 20 studies analyzing diagnostic accuracy, sensitivity was reported to be between 43% and 94.3%, specificity from 14% to 97.5%, and AUC from 0.640 to 0.980 [28]. In comparison, the combination model developed in this study demonstrated excellent diagnostic performance relative to these studies. Notably, a few prior studies on thyroid cancer have conducted additional validation tests to assess diagnostic accuracy following the initial AUC calculation with miRNA. A study analyzing PTC tissue using a panel of seven miRNAs reported a diagnostic accuracy of 95–98%, which exceeded that of our study [29]. However, FC studies analyzing miRNAs using subsequent validation tests, as conducted in this study, are rare. This scarcity is particularly evident in studies utilizing circulating miRNAs, rather than tissue-derived miRNAs. Therefore, the strength of our study lies in reevaluating the diagnostic accuracy of the developed model through additional validation testing, rather than relying on a single experimental outcome.

Limitations of this study include the single-center design and small FC sample size, especially in microarray to identify novel microRNA, restricting patient cohort diversity. This study was carried out over eleven years. Although the plasma samples were frozen and stored at −80 °C and our institution’s tissue bank conducts periodic QC evaluations to maintain sample quality, there were possible harmful effects on the quality of the microRNA material. This study did not adjust for sex and age-related miRNA expression variations. While miRNA levels may be influenced by these factors, analysis was not conducted due to sample size constraints. Additionally, the four-miRNA combination model exhibited high sensitivity and specificity, but decreased in validation testing. Further studies integrating miRNA levels with clinical findings, such as ultrasound and tumor size, are needed to improve FA-FC differentiation accuracy. RAS mutation is also an important factor that can affect the expression of miRNA [30]. However, this study did not analyze the expression of plasma miRNA according to RAS mutation. In the future, research on the expression pattern of plasma miRNA according to various mutations, including RAS mutation in follicular cell-derived thyroid cancer, is also necessary.

## 5. Conclusions

miR-6085 was identified as a novel plasma biomarker distinguishing FA from FC. Four circulating plasma microRNAs showed significant overexpression in FC compared to benign patients. The four-miRNA combination model (miR-6085, miR-146b-5p, miR-221, miR-222) demonstrated high diagnostic accuracy, sensitivity, and specificity for FC, supporting the potential utility of plasma microRNAs as diagnostic biomarkers. And these miRNAs can also be applied to target therapy in the future. For the clinical application of these miRNAs in the future, research on the mechanism of FC development and large-scale cohort studies is necessary.

## Figures and Tables

**Table 1:** Characteristics of total of 90 enrolled patients.

Characteristics	Utility Assessment (n = 58)			Validation Test (n = 32)		
	FA	FC	Overall	FA	FC	Overall
	(n = 25)	(n = 33)	(n = 58)	(n = 16)	(n = 16)	(n = 32)
Sex						
Male	5 (20.0%)	5 (17.25)	10 (17.2%)	3 (18.8%)	5 (31.2%)	8 (25.0%)
Female	20 (80.0%)	28 (84.8%)	48 (82.3%)	13 (81.2%)	11 (68.8%)	24 (75.0%)
Age (years, Median, IQR)	52.00 (41.00–64.00)	57.00 (44.00–65.00)	54.00 (44.00–64.00)	46.50 (44.00–63.00)	53.50 (41.75–62.75)	51.50 (43.25–63.25)
Tumor size (cm, Median, IQR)	2.50 (1.40–4.00)	3.30 (2.60–4.00)	3.00 (2.00–3.78)	2.05 (1.00–2.85)	3.00 (1.80–3.60)	2.45 (1.78–3.25)
Multifocality		0			2	
Lymph node metastasis		2			0	
Distant metastasis		1			0	
Aggressiveness						
Minimally invasive FC		25 (75.8%)			14 (87.5%)	
Widely invasive FC		8 (24.2%)			2 (12.5%)	

FA: follicular thyroid adenoma; FC: follicular thyroid carcinoma; Multifocality, lymph node metastasis, distant metastasis; the number of patients with the corresponding characteristics.

**Table 2:** Characteristics of 15 patients included in the microarray analysis.

Characteristics	FA (n = 5)	FC (n = 10)	Overall (n = 15)
Sex			
Male	1 (20%)	2 (20%)	3 (20%)
Female	4 (80%)	8 (80%)	12 (80%)
Age (years, Median, IQR)	48.00 (36.00–51.00)	48.50 (36.00–63.00)	48.00 (40.50–57.00)
Tumor size (cm, Median, IQR)	2.70 (2.10–3.50)	3.35 (2.60–3.90)	3.10 (2.60–3.75)
Multifocality		0	
Lymph node metastasis		2	
Distant metastasis		0	
Aggressiveness			
Minimally invasive FC	-	5 (50%)	
Widely invasive FC	-	5 (50%)	

FA: follicular thyroid adenoma; FC: follicular thyroid carcinoma; Multifocality, lymph node metastasis, distant metastasis; the number of patients with the corresponding characteristics.

**Table 3:** Plasma miRNAs most effective in differentiating follicular thyroid carcinoma from follicular thyroid adenoma.

miRNA Base	FC/FA Fold Change	FC/FA Raw *p*-Value
miR-6085	1.75	0.011
ENSG00000239080 (probe ID; 20533711)	−2.07	0.013
ENSG00000239080 (probe ID; 20533712)	−1.78	0.019

FA: follicular thyroid adenoma; FC: follicular thyroid carcinoma.

**Table 4:** Diagnostic models for predicting follicular thyroid carcinoma.

Model	miRNA	AUC (95% CI)	Sensitivity (95% CI)	Specificity (95% CI)	Cut-Off Value	BIC	Equation
1-1	miR-6085	0.702 (0.557, 0.848)	1.000 (1.000, 1.000)	0.360 (0.200, 0.560)	≥−0.267	69.07	Logit(P) = −0.384 + 1.335 × mRNA.6085_log
1-2	miR-146b-5p	0.735 (0.587, 0.883)	0.667 (0.458, 0.833)	0.840 (0.680, 0.960)	≥0.593	67.77	Logit(P) = −0.475 + 1.289 × mRNA.146b-5p_log
1-3	miR-221	0.654 (0.491, 0.817)	0.478 (0.261, 0.696)	0.913 (0.783, 1.000)	≥0.888	67.44	Logit(P) = −0.176 + 0.77 × mRNA.221_log
1-4	miR-222	0.877 (0.771, 0.983)	0.952 (0.857, 1.000)	0.708 (0.500, 0.875)	≥0.608	44.86	Logit(P) = −2.073 + 2.232 × mRNA.222_log
2-1	miR-221 + miR-222	0.902 (0.804, 1.000)	0.950 (0.850, 1.000)	0.864 (0.727, 1.000)	≥0.575	41.12	Logit(P) = −2.928 + 1.229 × mRNA.221_log + 2.676 × mRNA.222_log
2-2	miR-6085 + miR-222	0.901 (0.806, 0.996)	0.905 (0.762, 1.000)	0.792 (0.625, 0.958)	≥0.362	46.74	Logit(P) = −2.304 + 0.946 × mRNA.6085_log + 2.219 × mRNA.222_log
2-3	miR-146b-5p + miR-222	0.885 (0.787, 0.984)	0.900 (0.750, 1.000)	0.792 (0.625, 0.917)	≥0.382	47.33	Logit(P) = −2.118 + 0.524 × mRNA.146b-5p_log + 2.043 × mRNA.222_log
3-1	miR-6085 + miR-221 + miR-222	0.927 (0.840, 1.000)	0.900 (0.750, 1.000)	0.909 (0.773, 1.000)	≥0.59	42.18	Logit(P) = −3.579 + 1.474 × mRNA.6085_log + 1.4 × mRNA.221_log + 2.808 × mRNA.222_log
3-2	miR-146b-5p + miR-221 + miR-222	0.911 (0.823, 1.000)	0.947 (0.842, 1.000)	0.818 (0.636, 0.955)	≥0.487	43	Logit(P) = −3.091 + 0.827 × mRNA.146b-5p_log + 1.417 × mRNA.221_log + 2.443 × mRNA.222_log
3-3	miR-6085 + miR-146b-5p + miR-222	0.900 (0.806, 0.994)	0.900 (0.750, 1.000)	0.792 (0.625, 0.958)	≥0.366	49.74	Logit(P) = −2.314 + 1.042 × mRNA.6085_log + −0.049 × mRNA.146b-5p_log + 2.164 × mRNA.222_log
4	miR-6085 + miR-146b-5p + miR-221 + miR-222	0.928 (0.843, 1.000)	0.947 (0.842, 1.000)	0.864 (0.682, 1.000)	≥0.432	44.77	Logit(P) = −3.682 + 1.582 × mRNA.6085_log + 0.103 × mRNA.146b-5p_log + 1.468 × mRNA.221_log + 2.723 × mRNA.222_log

AUC: Area Under the Curve, BIC: Bayesian Information Criterion.

**Table 5:** Validation test of predictive models.

Model	miRNA	Accuracy (95% CI)	Sensitivity (95% CI)	Specificity (95% CI)
1-1	miR-6085	0.586 (0.389, 0.765)	0.500 (0.247, 0.753)	0.692 (0.386, 0.909)
1-2	miR-146b-5p	0.593 (0.388, 0.776)	0.643 (0.351, 0.872)	0.538 (0.251, 0.808)
1-3	miR-221	0.643 (0.441, 0.814)	0.857 (0.572, 0.982)	0.429 (0.177, 0.711)
1-4	miR-222	0.679 (0.476, 0.841)	0.769 (0.462, 0.950)	0.600 (0.323, 0.837)
2-1	miR-221 + miR-222	0.600 (0.387, 0.789)	0.727 (0.390, 0.940)	0.500 (0.230, 0.770)
2-2	miR-6085 + miR-222	0.538 (0.334, 0.734)	0.538 (0.251, 0.808)	0.538 (0.251, 0.808)
2-3	miR-146b-5p + miR-222	0.609 (0.385, 0.803)	0.545 (0.234, 0.833)	0.667 (0.349, 0.901)
3-1	miR-6085 + miR-221 + miR-222	0.739 (0.516, 0.898)	0.727 (0.390, 0.940)	0.750 (0.428, 0.945)
3-2	miR-146b-5p + miR-221 + miR-222	0.714 (0.478, 0.887)	0.700 (0.348, 0.933)	0.727 (0.390, 0.940)
3-3	miR-6085 + miR-146b-5p + miR-222	0.522 (0.306, 0.732)	0.455 (0.167, 0.766)	0.583 (0.277, 0.848)
4	miR-6085 + miR-146b-5p + miR-221 + miR-222	0.762 (0.528, 0.918)	0.700 (0.348, 0.933)	0.818 (0.482, 0.977)

## Data Availability

The data underlying this study are not publicly available due to privacy and ethical restrictions.
